# Mapping the Free Energy Landscape of PKA Inhibition and Activation: A Double-Conformational Selection Model for the Tandem cAMP-Binding Domains of PKA RIα

**DOI:** 10.1371/journal.pbio.1002305

**Published:** 2015-11-30

**Authors:** Madoka Akimoto, Eric Tyler McNicholl, Avinash Ramkissoon, Kody Moleschi, Susan S. Taylor, Giuseppe Melacini

**Affiliations:** 1 Department of Chemistry and Chemical Biology, McMaster University, Hamilton, Ontario, Canada; 2 Department of Biochemistry and Biomedical Sciences, McMaster University, Hamilton, Ontario, Canada; 3 Department of Chemistry and Biochemistry, Department of Pharmacology and Howard Hughes Medical Institute, University of California, San Diego, La Jolla, California, United States of America; Rutgers University, UNITED STATES OF AMERICA

## Abstract

Protein Kinase A (PKA) is the major receptor for the cyclic adenosine monophosphate (cAMP) secondary messenger in eukaryotes. cAMP binds to two tandem cAMP-binding domains (CBD-A and -B) within the regulatory subunit of PKA (R), unleashing the activity of the catalytic subunit (C). While CBD-A in RIα is required for PKA inhibition and activation, CBD-B functions as a “gatekeeper” domain that modulates the control exerted by CBD-A. Preliminary evidence suggests that CBD-B dynamics are critical for its gatekeeper function. To test this hypothesis, here we investigate by Nuclear Magnetic Resonance (NMR) the two-domain construct RIα (91–379) in its apo, cAMP_2_, and C-bound forms. Our comparative NMR analyses lead to a double conformational selection model in which each apo CBD dynamically samples both active and inactive states independently of the adjacent CBD within a nearly degenerate free energy landscape. Such degeneracy is critical to explain the sensitivity of CBD-B to weak interactions with C and its high affinity for cAMP. Binding of cAMP eliminates this degeneracy, as it selectively stabilizes the active conformation within each CBD and inter-CBD contacts, which require both cAMP and W260. The latter is contributed by CBD-B and mediates capping of the cAMP bound to CBD-A. The inter-CBD interface is dispensable for intra-CBD conformational selection, but is indispensable for full activation of PKA as it occludes C-subunit recognition sites within CBD-A. In addition, the two structurally homologous cAMP-bound CBDs exhibit marked differences in their residual dynamics profiles, supporting the notion that conservation of structure does not necessarily imply conservation of dynamics.

## Abstract 2

Protein Kinase A (PKA) is the major receptor for the cAMP secondary messenger in eukaryotes. This study shows how PKA's regulatory subunit dynamically samples a degenerate free energy landscape that controls affinities for the catalytic subunit and cAMP; intra-domain conformational selection by cAMP controls inter-domain interactions and PKA activation.

## Author Summary

Cyclic adenosine monophosphate (cAMP) is a messenger molecule produced within cells to control cellular metabolism in response to external stimuli. Protein Kinase A (PKA) is the major receptor for cAMP. cAMP binds to tandem cAMP-binding domains (CBD-A and -B) within the regulatory subunits of PKA (R), unleashing the activity of the catalytic subunit (C). While CBD-A is required for C-subunit inhibition and activation, in RIα CBD-B functions as a “gatekeeper” domain that modulates the control exerted by CBD-A. However, it is not currently clear how ligand binding and dynamics of CBD-B mediate its gatekeeper function. We comparatively analyzed by Nuclear Magnetic Resonance (NMR) a two-domain construct of the regulatory subunit RIα with no ligand, with cAMP_2_ bound, and the C-bound form. These data show that both CBDs can exist in a system of uncorrelated conformational selection as both can independently sample activated and inactivated states (in what is known as a nearly degenerate free energy landscape). This explains why both RIα CBDs exhibit a higher cAMP-affinity than other cAMP receptors. Once cAMP has bound, the degeneracy is lost and dissociation of the kinase subunit is promoted through a combination of intra-domain conformational selection and changes in inter-CBD orientation. The proposed model—a double-conformational selection model—provides a general framework to interpret the effect of PKA mutations that have been reported in rare human disorders such as Carney complex and Acrodysostosis.

## Introduction

**Fig 1:**
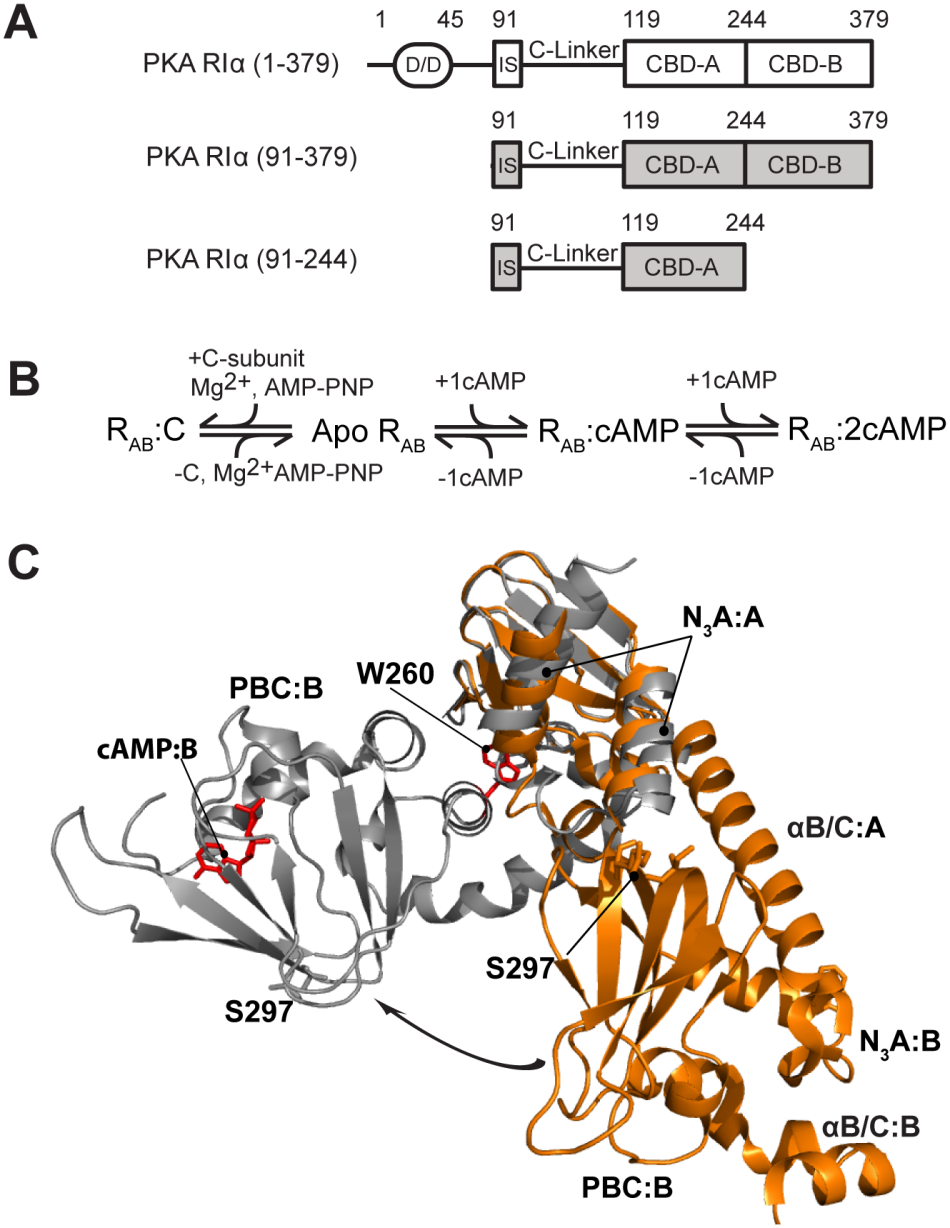
Construct design and architecture of PKA RIα. **(A)** Domain organization of the RIα subunit of PKA and its functional constructs that ensure cAMP-dependent inhibition of the catalytic subunit (C). **(B)** Binding equilibria of PKA RIα. Even when apo RIα is a transient low-population intermediate, it is still a critical thermodynamic determinant of the cAMP-dependent regulation of PKA. **(C)** Structures of PKA RIα (91–379) bound to either cAMP (grey, PDB code 1RGS) or the C-subunit (orange, PDB code 2QCS for the R333K mutant of RIα (91–379)). The two structures are superimposed through the β-barrel of cAMP-binding domain (CBD) A. Selected regions and residues are labeled. The arrow shows the change in the position of CBD-B relative to CBD-A occurring upon cAMP-binding.

Cyclic adenosine monophosphate (cAMP) is an ancient secondary messenger, and in higher eukaryotes, Protein Kinase A (PKA) is the major receptor for cAMP. The cAMP-dependent activation of PKA is utilized by a wide variety of extracellular stimuli to control the respective intra-cellular responses, such as regulation of the immune system and cell proliferation [1–4]. In the resting state, PKA exists as an inhibited tetramer formed by a dimeric regulatory subunit (R_2_), with each R-protomer binding and inhibiting one equivalent of catalytic subunit (C). Upon binding of four equivalents of cAMP to R_2_, the R_2_C_2_ tetramer at least partially dissociates, releasing the active C-subunit to phosphorylate downstream substrates [5–10]. The R-subunit of PKA is a multi-domain protein (Fig 1A), starting from a dimerization-docking domain followed by a flexible linker, which includes an inhibitory site for C and is in turn followed by two tandem cAMP-binding domains (CBD-A and -B) that provide additional contact sites for binding the C-subunit [11,12]. The RIα (91–379) construct spans the inhibitory site and both CBDs (Fig 1A) and recapitulates most of the features that are associated with both C-inhibition and cAMP-dependent activation of full-length PKA [13–15]. The structures of this construct have been solved in both the cAMP_2_-bound (i.e., active wild type [WT]) [14] and C-bound forms (i.e., inactive R333K mutant) [13], revealing major conformational changes that underlie the cAMP-dependent activation of PKA (Fig 1C). Furthermore, the dynamics of apo CBD-A have been recently shown to be another central determinant of the allosteric control of PKA activation by cAMP [16]. However, less is known about the dynamics of CBD-B and how they relate to the physiological function of this domain.

The CBD-B serves a pivotal function in both PKA inhibition and activation. The structure of C-bound RIα (91–379) has revealed that CBD-B contributes to the effective inhibition of the C-subunit, as the N-terminal helix of CBD-B provides a key site for binding C [13]. The structure of cAMP_2_-bound RIα (91–379), as well as mutagenesis and Markov state models, has shown that CBD-B also facilitates the cAMP-dependent release of C from R by functioning as a “gate-keeper” domain [10,17,18] that controls the availability of a Trp indole (i.e., W260), which stacks against the adenine of cAMP bound to CBD-A and acts as a lid for CBD-A [10,14,15,18,19]. However, as in the case of CBD-A [16], the cAMP- and C-bound structures of CBD-B are not sufficient for a full thermodynamic dissection of the central function of CBD-B, which requires also the investigation of apo CBD-B.

The currently available evidence suggests that apo CBD-B is highly dynamic. The structure of RIα (91–379) with cGMP bound to site A and an apo site B revealed overall elevated B-factors for CBD-B relative to CBD-A [20]. Furthermore, a small-angle X-ray scattering (SAXS) investigation of the R:C complex indicated that the orientation of CBD-B relative to CBD-A and the C-subunit is dynamic [21,22]. In addition, molecular dynamics (MD) simulations confirmed that the helix connecting CBD-A and -B is flexible in the absence of cAMP [23]. Overall, these initial results suggest that dynamics of nucleotide-free CBD-B are functionally critical, although currently the relationship between apo CBD-B dynamics and its function is not fully understood [24,25].

Here, we hypothesize that apo CBD-B pre-samples both active and inactive conformations with the binding of cAMP or the C-subunit selecting for the former or the latter, respectively. In order to test this hypothesis about conformational selection in CBD-B and to understand how the intra-CBD dynamics affect inter-CBD interactions, we comparatively analyzed by Nuclear Magnetic Resonance (NMR) the two-domain construct RIα (91–379) in its apo, cAMP_2_, and C-bound forms. The latter is the most technically challenging form of RIα (91–379) both in terms of sample preparation and NMR data acquisition [26–31], given the high MW (i.e., ~73 kDa), and was lacking in previous NMR investigations [32,33]. However, C-bound RIα (91–379) is included here because it plays a critical role in our comparative NMR analysis scheme as an essential reference term for the inactive state of R. In addition, the present comparative analyses include mutations designed to specifically probe inter-domain interactions [34,35] and provide an opportunity to re-assess previous results on CBD-A [16,36] in the context of a more complete construct with both tandem CBD-A and -B.

Our comparative NMR analysis supports a model of double conformational selection in which each apo CBD populates a nearly degenerate free energy landscape independently of the adjacent CBD. Such degeneracy is unusual for allosteric systems, which typically sample highly skewed conformational equilibria in their apo form [37–42], but it explains why even weak interactions with the C-subunit are sufficient for conformational selection, as well as why both RIα CBDs bind cAMP with higher affinity than other eukaryotic CBDs. Binding of two equivalents of cAMP eliminates this degeneracy by selecting the active conformation within each CBD and stabilizing the inter-CBD contacts. We also show that the CBD-A/B interface requires both cAMP and the indole of W260 to form, and, although it is not required for the intra-domain conformational selection, it contributes to the activation of PKA by occluding C-subunit recognition sites within CBD-A. Hence, the proposed model explains how CBD-B contributes to PKA inhibition and activation. In addition, our investigation revealed that cAMP-binding does not fully quench the dynamics of the CBDs, with major loci of residual dynamics found both at the inter-CBD interface and within each tandem CBD. Surprisingly, the residual dynamics profiles of the structurally homologous CBD-A and -B are different. This unexpected finding supports the notion that conservation of structure does not necessarily imply conservation of dynamics and opens new opportunities in the selective targeting of CBDs for therapeutic purposes.

## Results

### Intra-Domain Conformational Selection in PKA RIα CBDs

**Fig 2:**
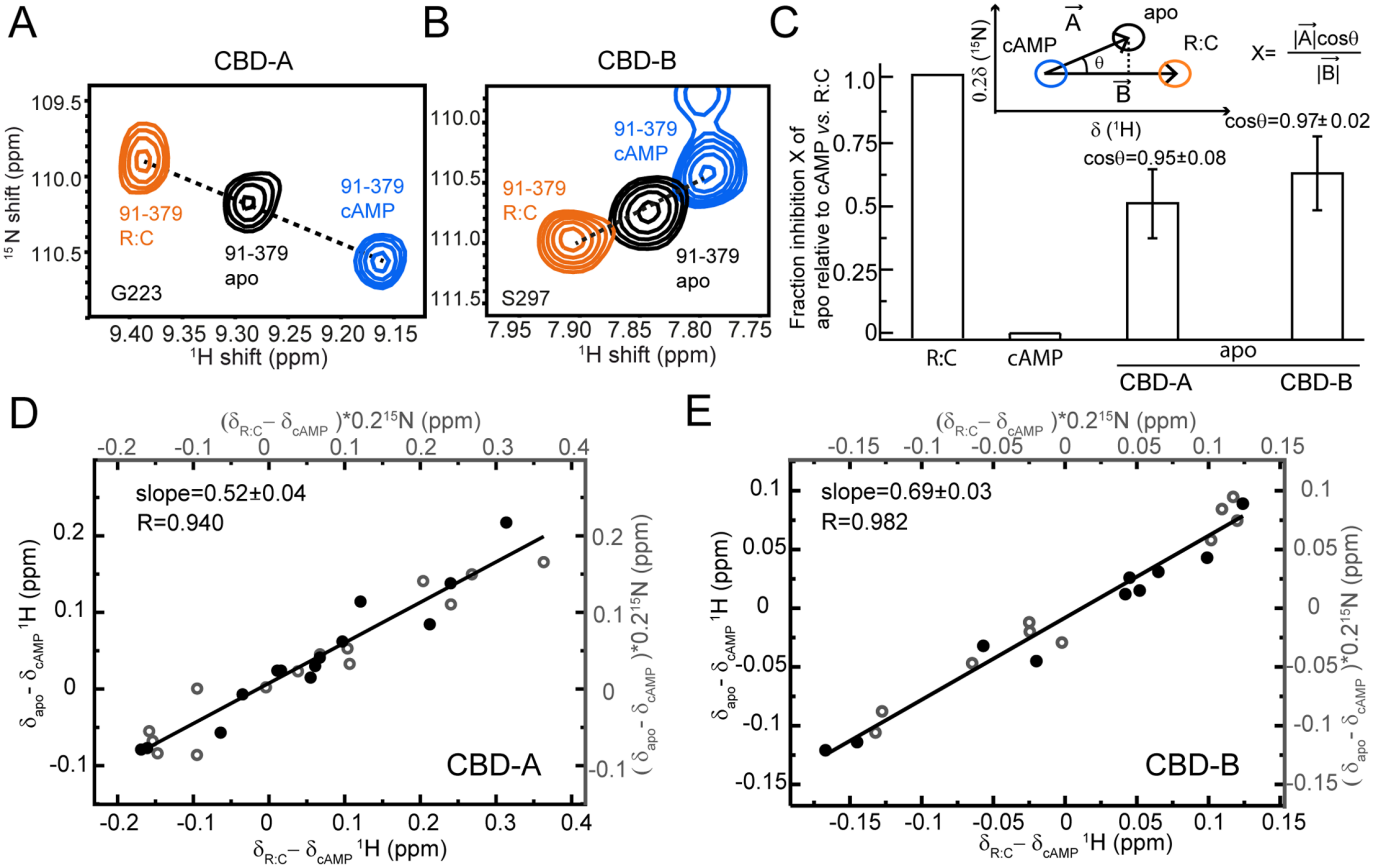
Conformational selection in CBD-A and -B of RIα (91–379). **(A, B)** Overlay of apo, cAMP_2_-bound, and C-bound RIα (91–379) for cross-peaks of representative residues sensing the active versus inactive equilibria in CBD-A (A) and CBD-B (B). **(C)** Fractional inhibition of apo RIα (91–379) (X) relative to cAMP_2_- and C-bound RIα (91–379), assuming they represent the active and inactive forms of PKA, respectively. The inset illustrates how X was measured using the CHEmical Shift Projection Analysis (CHESPA) method. The bar graph shows the average X values observed for residues in CBD-A and -B exhibiting a linear pattern (|cosθ| > 0.95). **(D)** Alternative evaluation of the fractional inhibition of apo RIα (91–379) (X) through the slope of the (δ_apo−_δ_cAMP_) versus (δ_C−_δ_cAMP_) plot, where δ_apo_, δ_cAMP_, and δ_C_ are the chemical shifts of apo, cAMP_2_- and C-bound RIα (91–379). Closed and open circles indicate ^1^H and ^15^N chemical shifts, respectively. This plot was restricted to CBD-A residues that are sufficiently removed from the cAMP-dependent interfaces (e.g., R:C, R:cAMP and CBD-A:CBD-B) to report primarily on the active versus inactive equilibrium of CBD-A. **(E)** Similar to panel (D) but for CBD-B.

As a first step towards mapping the functional conformational equilibria of apo PKA RIα (91–379), we compared it to two reference states, i.e., C-bound RIα (91–379), which is assumed to trap the inhibitory (“inactive”) conformation of RIα (91–379), and cAMP_2_-bound RIα (91–379), which is expected to represent the uninhibited (“active”) form of PKA-R. The comparative spectral analysis of apo versus C- versus cAMP_2_-bound RIα (91–379) is illustrated in Fig 2. Panels 2A and 2B focus on two representative residues of CBD-A and -B, i.e., G223 and S297, respectively, which are sufficiently removed from the cAMP and C-subunit binding interfaces to report primarily on the conformational equilibria of RIα (91–379). For both residues, the cross-peaks arising from the apo, C, and cAMP_2_-bound forms define a clear linear pattern, which is indicative of apo RIα (91–379) sampling two states (i.e., the active and inactive conformations) through an exchange that is fast in the chemical shift NMR time scale at the field of data acquisition (i.e., 700 MHz).

Given the fast regime for the active versus inactive exchange, the apo cross-peak position is simply a population-weighed linear average of the chemical shifts for the active and inactive conformations. Hence, the chemical shifts encode directly the relative populations of the active versus inactive equilibrium. For example, the intermediate position of the apo peaks of G223 and S297 relative to those of the C and cAMP_2_-bound forms (Fig 2A and 2B) suggests that in each apo CBD the populations of active and inactive states are comparable. In order to verify that this result does not depend on the specific choice of reporter residues (e.g., G223 and S297), the apo CBD active versus inactive populations were also assessed through the CHEmical Shift Projection Analysis (CHESPA) (Fig 2C) [37] and through complementary chemical shift correlations (Fig 2D and 2E). Both approaches rely on a wider collection of conformational equilibria-sensing residues as opposed to only one residue per CBD, as shown in Fig 2A and 2B.

The CHESPA analysis (Fig 2C) shows that, if C- and cAMP_2_-bound RIα (91–379) represent the inactive and active states of PKA, the average fractional activation observed for CBD-A is ~50%, with slightly higher values measured for CBD-B. The differences in average fractional activations between CBD-A and -B are not major as they are within one standard deviation of both distributions (Fig 2C). Similar conclusions are obtained through chemical shift correlation analyses (Fig 2D and 2E). Panels 2D and 2E report the (δ_Apo −_ δ_cAMP_) versus (δ_C_ − δ_cAMP_) chemical shift correlations for apo CBD-A and -B, respectively. The linearity of the correlations in Fig 2D and 2E confirms that each CBD samples a dynamic two-state equilibrium of active and inactive conformations. In addition, the slopes of the correlations provide the molar fraction of the inactive state, i.e., ~50% and ~70% for CBD-A and -B, respectively, corroborating that each apo CBD accesses significant populations of both inactive and active conformations, although the former appears slightly higher for CBD-B than CBD-A. However, the somewhat higher population of inactive state in apo CBD-B versus apo CBD-A may also reflect the fact that in the R:C complex the CBD-B does not interact with the C-subunit as tightly as CBD-A. The transient nature of the CBD-B interactions with the C-subunit may shift the R:C cross-peaks of CBD-B towards the apo cross-peaks, resulting in an increase in the apparent fractional inactivation measured for apo CBD-B. In this respect, the actual population of inactive state in apo CBD-B is possibly lower than the measured 70%, confirming that the CBD-B samples significant (i.e., >30%) populations of active state even prior to cAMP binding.

**Fig 3:**
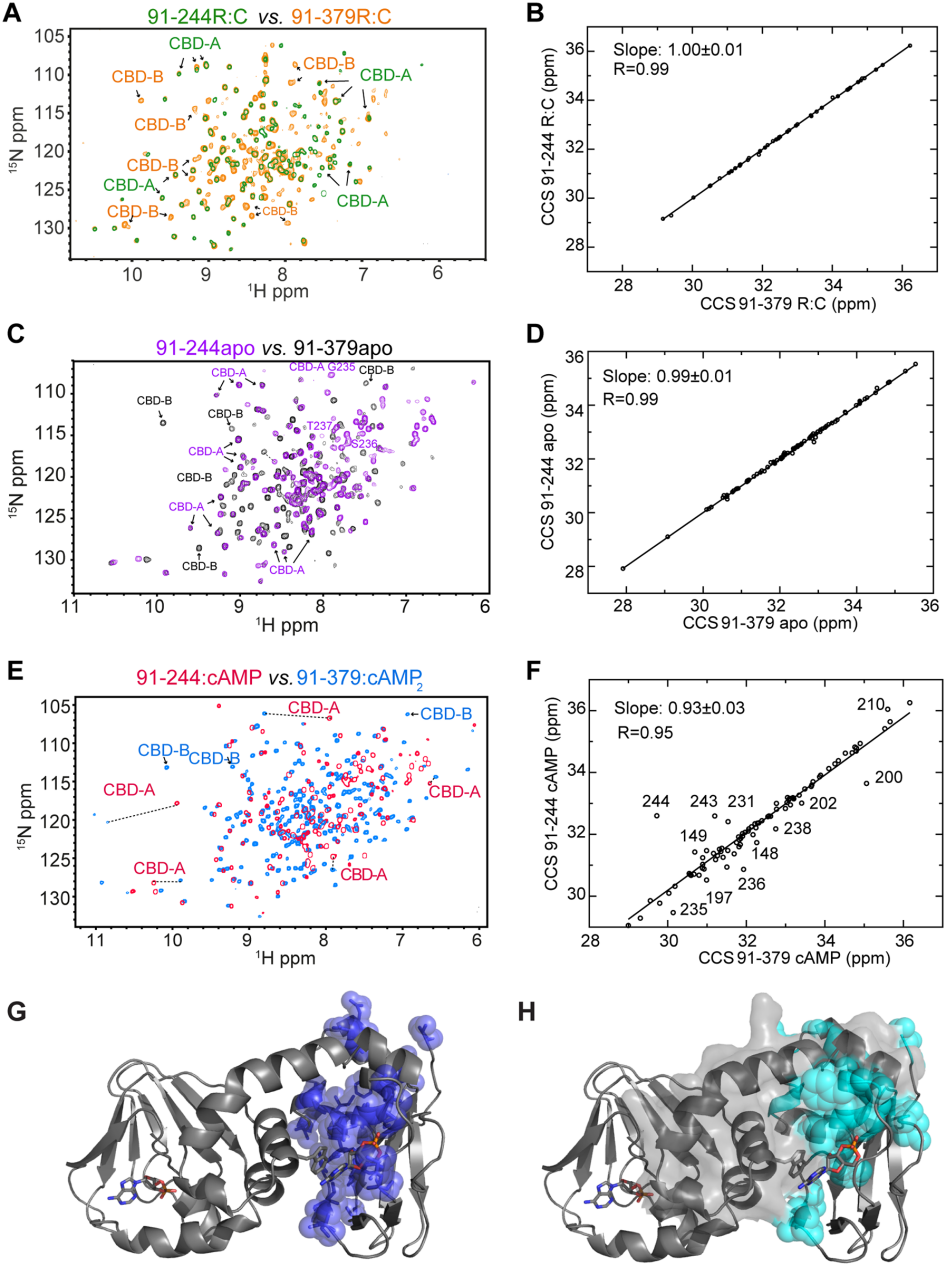
Probing CBD-A/B interactions in apo, C-bound, and cAMP_2_-bound RIα (91–379) through CBD-B deletion. **(A)** Overlay of the HN-TROSY spectra of C-bound RIα (91–379) and RIα (91–244), in which CBD-B is deleted. **(B)** Correlation between the combined chemical shifts (CCS) of C-bound RIα (91–379) and RIα (91–244). **(C, D)** As in panels (A, B), but for the apo forms of RIα (91–379) and RIα (91–244). **(E, F)** As in panels (A, B), but for the cAMP_2_-bound form of RIα (91–379) and the cAMP-bound form of RIα (91–244). Color codes for panels A, C, and E are indicated in the figure and representative CBD-A and -B cross-peaks are labeled. **(G)** Map of above-average RIα (91–379):cAMP_2_ versus RIα (91–244):cAMP CCS differences for residues <226 (blue spheres) onto the structure of RIα (91–379):cAMP_2_ (PDB Code: 1RGS). **(H)** Cyan spheres represent CBD-A residues experiencing solvent accessible surface area (SASA) changes upon deletion of residues 226–379, which are highlighted with a grey surface. This SASA map was built using the same structure as in panel (G).

It should be noted that even if we knew the exact value for the fractional inactivation of CBD-B, the data in Fig 2 alone would not be sufficient to gauge the degree of correlation between the active–inactive conformational transitions in the two tandem domains. For example, if the actual fractional inactivation of each CBD is 50%, two markedly different distributions of state populations are still possible: (1) 50% of active-active state, 50% of inactive-inactive state, and no mixed (inactive-active and active-inactive) states, or (2) 25% of each possible state. In the two scenarios the degree of correlation between the active-inactive transitions in the two domains is clearly different, but in both cases the fractional inactivation for each CBD is 50%. Hence, it is clear that understanding how the inactive versus active intra-CBD equilibria are coupled to each other requires an independent assessment of the CBD-A/B interactions in the context of a combinatorial analysis of CBD states (i.e., active-active, inactive-active, active-inactive, and inactive-inactive CBD-CBD states). For this purpose, we dissected the state-specificity of inter-domain interactions by probing the effect of CBD-B deletion on CBD-A, i.e., by comparing the RIα (91–379) versus RIα (91–244) constructs (Fig 3).

### State-Specific Inter-Domain Interactions As Mapped by CBD-B Deletion

Fig 3 reports on the comparative NMR analysis of the RIα (91–379) versus RIα (91–244) constructs in their apo versus C- versus cAMP_2_-bound forms. Fig 3A shows that the cross-peak positions of the RIα (91–244):C complex do not change significantly relative to RIα (91–379):C, which includes the CBD-B as well, suggesting that inter-CBD interactions are negligible in the C-bound state. This conclusion is also supported by the chemical shift similarity between the RIα (91–244):C and RIα (91–379):C complexes (Fig 3B). Considering that the C-subunit of PKA is selective for the inactive-state of both CBDs, panels 3A and 3B point to negligible inter-CBD interactions when both CBDs adopt the inactive conformation (“inactive-inactive” state).

Fig 3C and 3D re-examines the RIα (91–379) versus RIα (91–244) comparison shown in panels 3A and 3B, but in the absence of both C-subunit and cAMP, i.e., for the apo forms of both constructs. Again the majority of the residues of CBD-A do not experience significant chemical shift changes upon deletion of CBD-B (Fig 3C and 3D), suggesting negligible inter-CBD interactions in apo RIα (91–379). Considering that each CBD in apo RIα (91–379) samples comparable populations of the respective active and inactive states, the absence of detectable inter-CBD contacts in apo RIα (91–379) suggests that in the absence of cAMP inter-CBD interactions are negligible not only in the “inactive-inactive” state but also in the remaining three combinations, i.e., the “active-inactive,” the “inactive-active,” and the “active-active” states. Overall, our data indicate that CBD-A and -B are largely independent of each other when cAMP is absent.

Unlike the cases of apo or C-bound RIα (91–379) (Fig 3A–3D), in the presence of excess cAMP the deletion of CBD-B:cAMP causes extensive chemical shift changes in CBD-A:cAMP (Fig 3E and 3F), pointing to the presence of significant inter-domain interactions. Interestingly, the residues in CBD-A:cAMP experiencing chemical shift variations upon deletion of CBD-B:cAMP (Fig 3G) are similar to those subject to solvent accessible surface area (SASA) variations upon deletion of RIα (226–379), which includes CBD-B (Fig 3H). The similarity of the residue maps in panels 3G and 3H suggests that the chemical shift differences observed in Fig 3E and 3F reflect primarily the disruption of inter-domain contacts upon CBD-B:cAMP deletion as opposed to possible changes in the intra-CBD active versus inactive equilibria. Based on this result, we hypothesized that the contribution of domain-domain interactions to the conformational selection of the active state in both CBDs is negligible compared to the contribution of cAMP. In order to test this hypothesis, we further probed the inter-domain interactions within RIα (91–379):cAMP_2_ through the W260A point mutation. W260 belongs to CBD-B but, through aromatic stacking, contacts cAMP bound to CBD-A (Fig 1C). Hence, W260 is expected to play a pivotal role in the cAMP-dependent CBD-A/B interactions [13].

### The W260A Point Mutation Is Sufficient to Disrupt the CBD-A/B Interface

**Fig 4:**
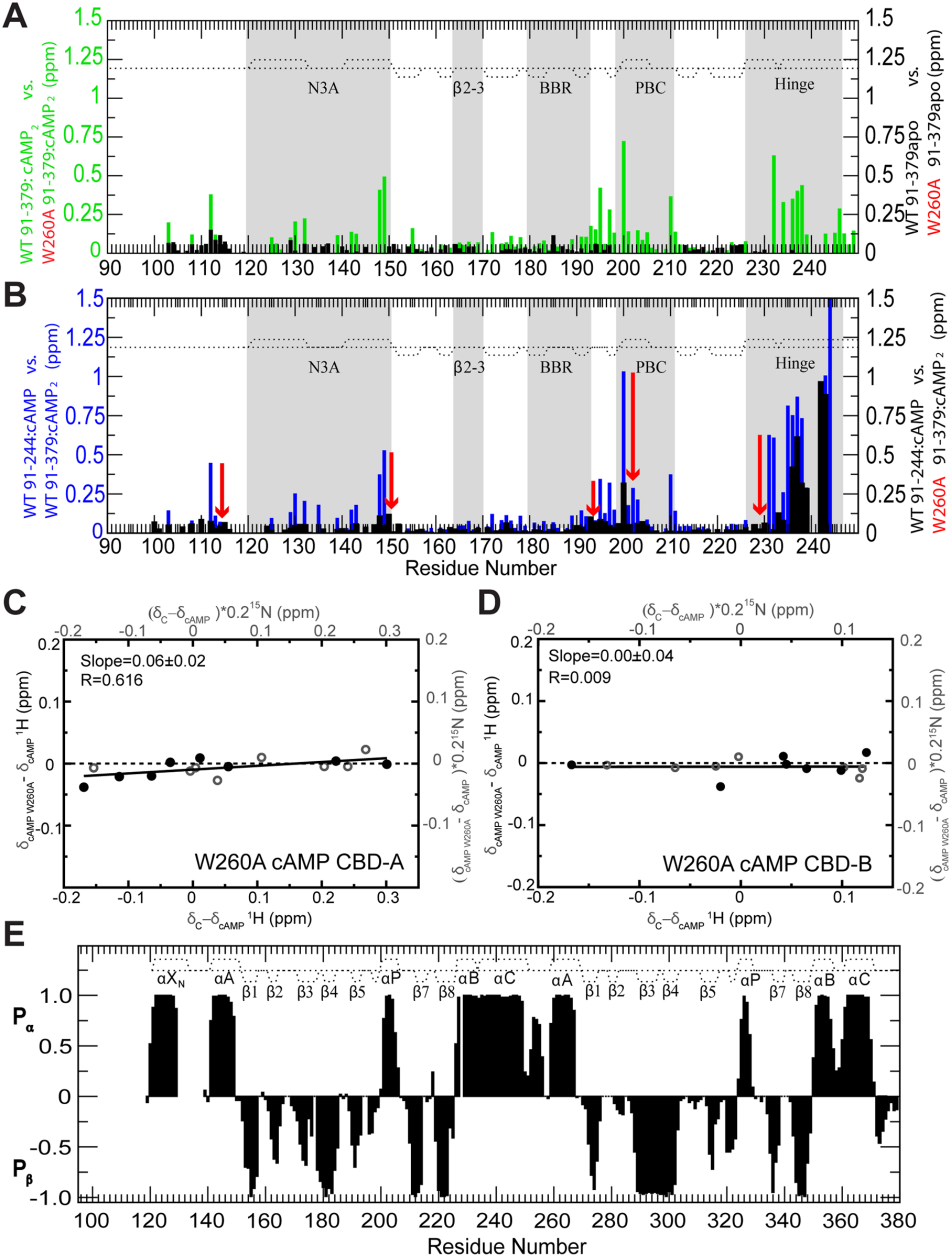
Probing CBD-A/B interactions in RIα (91–379) through the W260A mutation. Evaluation of the effect of the W260A mutation on the CBD-A/B interface through CCS differences in the apo and cAMP-bound states. **(A)** CCS changes caused by the W260A mutation in RIα (91–379) apo (black) or cAMP_2_-bound (green). **(B)** Plot of WT cAMP-bound RIα (91–244) versus WT cAMP_2_-bound RIα (91–379) CCS differences (blue) and plot of the WT cAMP-bound RIα (91–244) versus W260A cAMP_2_-bound RIα (91–379) CCS differences (black). The red arrows illustrate the effect of the W260A mutation. The comparison of the black and blue CCS difference profiles shows that the W260A mutation mimics the deletion of CBD-B. **(C)** Effect of the W260A mutation on the active versus inactive equilibrium of CBD-A, as assessed by chemical shift correlations, similarly to Fig 2D but with apo WT RIα (91–379) replaced by cAMP_2_-bound W260A RIα (91–379). **(D)** As in panel (C), but for CBD-B. **(E)** 2^ary^ structure probability map based on the secondary chemical shifts of the W260A mutant RIα (119–379):cAMP_2_ (black bars). The 2^ary^ structure profile of the WT:cAMP_2_ construct is reported as dotted lines.

**Fig 5:**
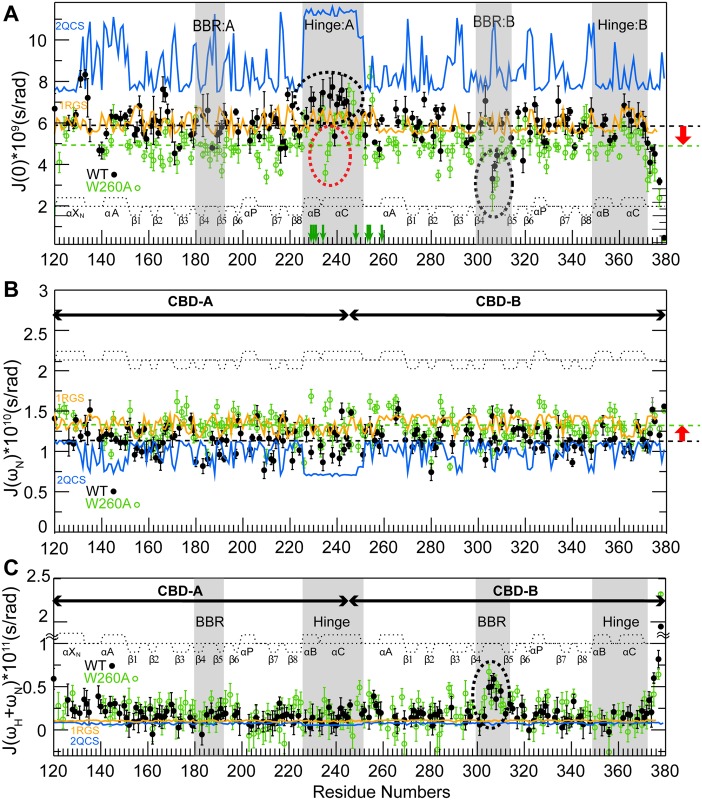
Intra- and inter-domain RIα dynamics. Dynamics of WT (black closed circles) and W260A (green open circles) RIα (119–379):cAMP_2_ mapped through residue-specific reduced spectral densities at 0 Hz, ω_N_, and ω_N_ + ω_H_ frequencies in panels **(A–C)**, respectively. Green vertical arrows in panel (A) mark residues of the helical region between the two β-barrels that are subject to fast H/D exchange (within the dead time) in W260A but not in the WT sample. The reduced spectral densities predicted based on the cAMP_2_- and C-bound RIα structures in the absence of internal motions are shown in orange and blue, respectively. The PDB codes of the structures used for the reduced spectral densities prediction are reported in the figure. Key regions subject to significant CBD-A versus B differences in dynamic profiles are highlighted in grey and with dashed ovals. The dashed horizontal lines in panels (A, B) indicate the average reduced spectral density values at 0 Hz and at ω_N_ for the WT (black) and W260A (green) constructs.

The W260A mutation causes major chemical shift changes in the presence of excess cAMP (Fig 4A, green bars) and these changes mimic those caused by the deletion of the whole CBD-B (Fig 4B). These chemical shift patterns suggest the single point W260A mutation is sufficient to disrupt the CBD-A/B interface. The disruption of the CBD-A/B interface caused by the W260A mutation was also independently confirmed through ^15^N-relaxation measurements, which were analyzed in terms of reduced spectral densities (Fig 5). The W260A mutation causes a decrease in the average J(0) value and a concurrent increase in the average J(ω_N_) value (Fig 5A and 5B, red vertical arrows; Table 1), which are indicative of a reduction of the effective correlation time for overall tumbling, as expected upon de-correlation of the tumbling motions of the two adjacent CBDs, i.e., the W260A mutant conforms better than WT to a model of two domains joined by a flexible linker. This result is independently confirmed by the observation that W260A enhances the flexibility of the helical region connecting the two tandem CBDs, as supported by the fact that several residues in this region experience a reduction of J(0) values (Fig 5A, red dashed oval) and an enhanced solvent exposure (Fig 5A, green arrows) due to the W260A mutation. Hence, the changes in dynamics caused by W260A corroborate the chemical shift perturbation results (Fig 4A and 4B), indicating that the W260A mutation is sufficient to disrupt the CBD-A/B interface.

**Table 1:** Spectral density mapping statistics^a^,^b^.

		**CBD-A**					
		**N3A**	**β2–3**	**BBR**	**PBC**	**Hinge**	**Total**
**WT**	J(0) × 10^9^ (s/rad)	6.13 ±0.20	6.29±0.38	5.90±0.53	5.81±0.52	**6.80±0.53**	**6.06±0.43**
	J(ω_N_) × 10^10^(s/rad)	1.19±0.07	1.12±0.08	1.00±0.07	1.12±0.08	1.14±0.08	**1.13±0.08**
	J(ω_H_+ω_N_) × 10^11^(s/rad)	0.26±0.08	0.14±0.09	0.15±0.09	0.09±0.10	0.15±0.10	0.16±0.10
**W260A**A	J(0) × 10^9^ (s/rad)	5.58±0.30	4.86±0.31	4.80±0.34	4.92±0.34	5.87±0.36	**5.07±0.33**
	J(ω_N_) × 10^10^ (s/rad)	1.21±0.06	1.25±0.08	1.29±0.07	1.35±0.07	1.27±0.08	**1.30±0.08**
	J(ω_H_+ω_N_) × 10^11^ (s/rad)	0.26±0.08	0.22±0.10	0.21±0.11	0.07±0.11	0.14±0.12	0.19±0.11
**2QCS** ^b^	J(0) × 10^9^ (s/rad)	8.88±1.29	8.75±1.01	8.66±1.13	8.27±0.68	11.19±0.41	8.95±1.42
	J(ω_N_) × 10^10^ (s/rad)	0.96±0.15	0.96±0.12	0.97±0.13	1.01±0.09	0.72±0.03	0.95±0.16
	J(ω_H_+ω_N_) × 10^11^(s/rad)	0.07±0.01	0.07±0.01	0.08±0.01	0.08±0.01	0.06±0.00	0.07±0.01
**1RGS** ^b^	J(0) × 10^9^ (s/rad)	5.91±0.32	5.88±0.33	6.19±0.45	5.83±0.30	6.13±0.32	6.01±0.39
	J(ω_N_) × 10^10^ (s/rad)	1.35±0.07	1.35±0.08	1.29±0.10	1.37±0.07	1.30±0.07	1.33±0.09
	J(ω_H_+ω_N_) × 10^11^ (s/rad)	0.11±0.01	0.11±0.01	0.10±0.01	0.11±0.01	0.10±0.01	0.10±0.01
		**CBD-B**					
		**N3A**	**β2–3**	**BBR**	**PBC**	**Hinge**	**Total**
**WT**	J(0) × 10^9^ (s/rad)	5.88±0.30	5.88±0.35	**5.30±0.36**	5.95±0.45	6.01±0.49	**5.63±0.37**
	J(ω_N_) × 10^10^ (s/rad)	1.26±0.08	1.17±0.09	1.19±0.08	1.14±0.09	1.12±0.08	**1.17±0.08**
	J(ω_H_+ω_N_) × 10^11^ (s/rad)	0.21±0.09	0.16±0.11	**0.33±0.09**	0.19±0.11	0.15±0.09	0.23±0.10
**W260A**	J(0) × 10^9^ (s/rad)	5.12±0.35	4.85±0.35	4.40±0.27	4.88±0.35	5.32±0.33	**4.87±0.34**
	J(ω_N_) × 10^10^ (s/rad)	1.39±0.07	1.38±0.08	1.24±0.06	1.35±0.07	1.26±0.08	**1.28±0.07**
	J(ω_H_+ω_N_) × 10^11^ (s/rad)	0.18±0.11	0.10±0.13	0.37±0.08	0.20±0.11	0.05±0.12	0.23±0.10
**2QCS** ^b^	J(0) × 10^9^ (s/rad)	8.98±1.45	8.16±0.73	8.33±0.87	8.30±0.96	8.04±0.60	8.49±1.11
	J(ω_N_) × 10^10^ (s/rad)	0.95±0.16	1.04±0.10	1.01±0.10	1.02±0.12	1.05±0.08	1.00±0.13
	J(ω_H_+ω_N_) × 10^11^ (s/rad)	0.07±0.01	0.08±0.01	0.08±0.01	0.08±0.01	0.08±0.01	0.08±0.01
**1RGS** ^b^	J(0) × 10^9^ (s/rad)	5.88±0.37	5.78±0.21	5.89±0.35	5.79±0.36	6.11±0.46	5.87±0.38
	J(ω_N_) × 10^10^ (s/rad)	1.36±0.08	1.38±0.05	1.36±0.08	1.38±0.08	1.31±0.10	1.36±0.09
	J(ω_H_+ω_N_) × 10^11^ (s/rad)	0.11±0.01	0.11±0.00	0.11±0.01	0.11±0.01	0.10±0.01	0.11±0.01

^***a***^CBD-A includes residues 119–268, while CBD-B spans residues 244–379. Spectral density averages were computed for major allosteric or binding hot spots, which include the N3A motif (residues 119–150), the β2–3 loop (residues 163–171), the BBR (residues 180–193), the PBC (residues 199–211), and the hinge (residues 226–251) in CBD-A. For CBD-B, the residue boundaries are: N3A motif (residues 244–268, which includes the W260 lid for CBD-A), β2–3 (residues 281–289), BBR (residues 298–316), PBC (residues 323–335), and hinge (residues 350–370). Average values which are more critical for the discussion (see text) are highlighted in bold.

^*b*^Spectral density values for RIα (119–379) either bound to the C-subunit (PDB Code: 2QCS) or to two equivalents of cAMP (PDB Code: 1RGS) were computed using HydroNMR, which models only the overall tumbling as opposed to internal motions.

### Dynamics at the CBD-A/B Junction

The observation that a single point mutation (i.e., W260A) compromises the integrity of a whole inter-CBD surface is suggestive of weak inter-CBD interactions, even in the presence of cAMP. In order to further investigate this hypothesis, we inspected the dynamics of the B-C hinge helices, which join CBD-A to CBD-B. As shown in Fig 5A, these helices exhibit J(0) values that are higher than other regions of the two-domain construct, suggesting the presence of residual ms-μs dynamics or diffusional anisotropy in the inter-domain hinge region even in the presence of excess cAMP. Diffusional anisotropy contributions arise in principle from either the main conformation, represented by the structure of cAMP_2_-bound RIα [14] with the two CBDs in close contact (Fig 1C), or possibly from minor populations of less compact and highly anisotropic conformers in which the CBD contacts are lost, as in the case of the dumbbell-shaped structure of C-bound RIα [13].

The diffusional anisotropy of both types of conformations was assessed through hydrodynamic simulations (Fig 5A, orange and blue traces) and in both cases a negligible contribution of diffusional anisotropy was determined for the elevated J(0) values observed in the B-C hinge helices connecting CBD-A to CBD-B. The observed J(0) values in the CBD-A hinge region are higher than the J(0) values predicted by hydrodynamic modeling based on the structure of the cAMP_2_-bound two-domain construct in the absence of internal motions (Fig 5A, orange trace; Table 1). Hence, the anisotropy of this compact conformer cannot account for the J(0) values observed in the hinge:A region (Fig 5A). Furthermore, although the diffusional anisotropy is amplified in the dumbbell-shaped structure observed for the C-bound R-subunit (Fig 5A, blue trace), contributions from this type of conformation to the elevated J(0) values observed for the hinge region of CBD-A are unlikely, as other regions (e.g., β8 of CBD-B) should then also display elevated J(0) values, which is not the case (Fig 5A). These considerations confirm that the elevated J(0) values observed for the B-C hinge helices connecting CBD-A to CBD-B reflect primarily ms-μs residual dynamics, which remain even after cAMP-binding to both domains (Fig 5A). The dynamic nature of the B-C helices joining CBD-A and -B calls for an assessment of the role of inter-domain interactions on the intra-CBD conformational equilibria.

### The Contribution of CBD-A/B Interactions to the Intra-CBD Conformational Selection Is Marginal

The effect of inter-domain interactions on the conformational equilibria of each CBD was probed using the W260A mutation to selectively disrupt the CBD-A/B interface without significantly affecting the structures of the two CBDs, which are largely unperturbed by the mutation (Fig 4E). Specifically, we measured the degree of correlation between the W260A:cAMP_2_ versus WT:cAMP_2,_ and the WT:C versus WT:cAMP_2_ chemical shift differences (Fig 4C and 4D). Similarly to the correlations shown in Fig 2D and 2E, this plot was restricted to residues that are sufficiently removed from the cAMP-dependent interfaces (e.g., R:C, R:cAMP, and CBD-A:CBD-B) to report primarily on the active versus inactive equilibrium of each CBD. However, unlike Fig 2D and 2E, no significant correlation was observed in either CBD-A or -B (Fig 4C and 4D), confirming the hypothesis that the contribution of inter-CBD interactions to the intra-CBD conformational selection is marginal compared to the contribution of cAMP (Fig 2). This hypothesis is also independently confirmed by previous results based on the RIα (91–244) CBD-A construct, showing linear cross-peak patterns with the apo form found in a central position, as in Fig 2A for CBD-A in the two-domain construct [16]. Overall, both the W260A mutation and the CBD-B deletion consistently indicate that cAMP-binding alone is sufficient to almost quantitatively stabilize the active-state of each CBD, even in the absence of CBD-CBD contacts. However, cAMP-binding does not result in a full quenching of dynamics and the profile of residual dynamics after cAMP binding defines critical differences between the two structurally homologous CBDs.

### A Versus B Differential CBD:cAMP Dynamics

The ms-μs residual dynamics detected for the B-C helices is a unique feature of CBD-A, as no noticeable internal dynamics were observed for the B-C helices of CBD-B (Fig 5A). In fact, both J(0) and J(ω_H_+ω_N_) spectral densities measured for residues in the B-C helices of CBD-B appear in overall good agreement with the values predicted based on hydrodynamic simulations starting from the cAMP_2_-bound structure of the R-subunit in the absence of internal motions (Fig 5A and 5C). Hence, the hinge helix dynamic profile appears to be highly domain-specific. Another region subject to a domain-specific dynamic profile is the base-binding region (BBR). The BBR of CBD-B exhibits elevated J(ω_H_+ω_N_) values relative to other regions of CBD-B, with the exception of the C-terminal tail (Fig 5C and Table 1), indicating that the BBR:B is flexible in the ps-ns time-scale. Unlike BBR:B, for BBR:A no internal dynamics were detected, as both J(0) and J(ω_H_+ω_N_) spectral densities for BBR:A residues appear in agreement with the values expected based on rigid-body hydrodynamic modeling (Fig 5A and 5C; Table 1). Overall, distinct dynamic profiles emerge for CBD-A and -B, in spite of their structural homology. The B-C helices exhibit ms-μs flexibility in CBD-A but not in CBD-B, while the BBR region is subject to ps-ns dynamics in CBD-B but not in CBD-A (Fig 5 and Table 1).

## Discussion

### A “Double Conformational Selection” Model for the cAMP-Dependent Control of the Inhibitory Interactions Mediated by the CBDs of PKA-RIα

**Fig 6:**
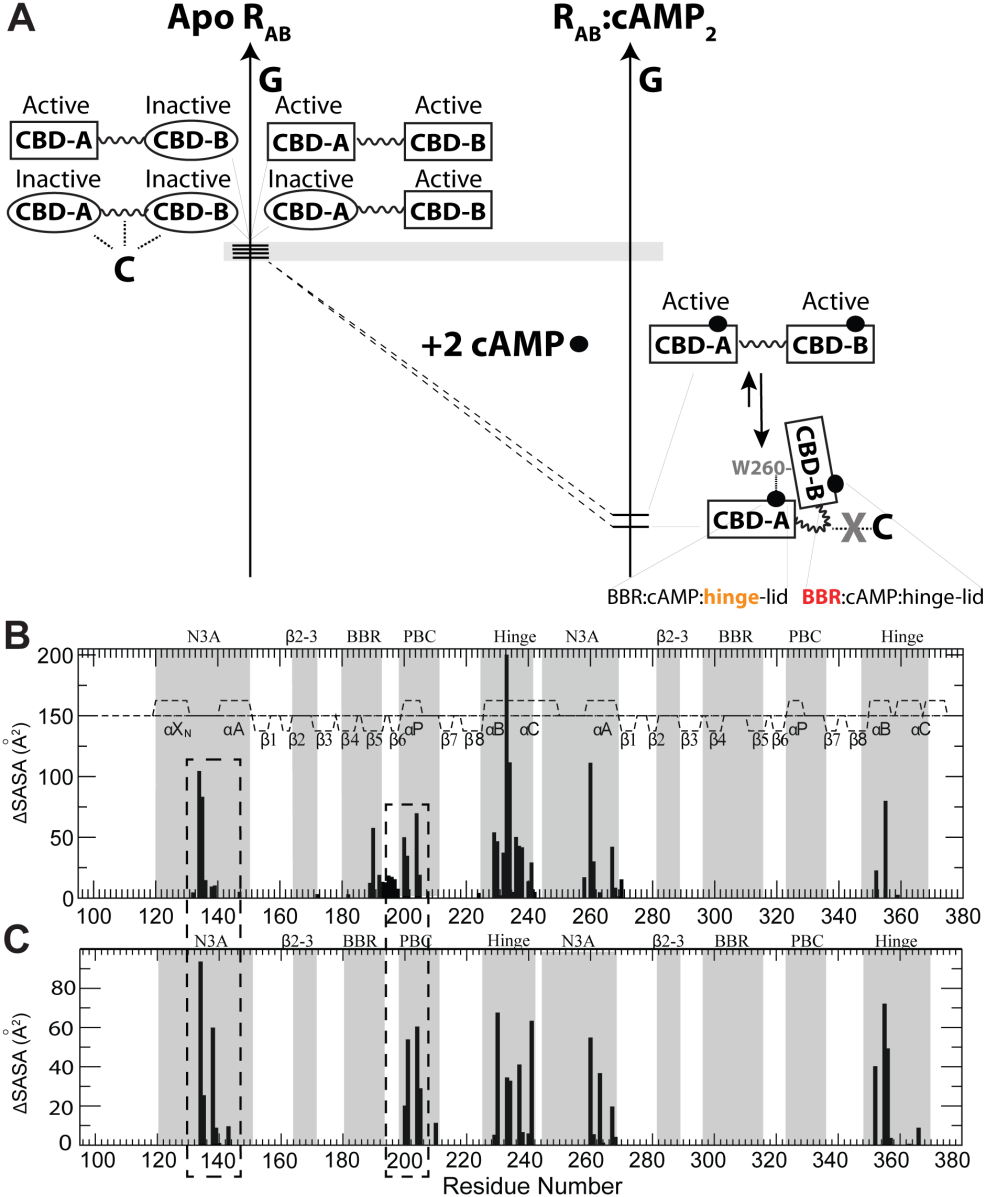
**(A)** “Double Conformational Selection” Model proposed for PKA-RIα activation. The inactive and active states of each CBD are represented through ovals and rectangles, respectively. The grey horizontal bar indicates that free energy differences are minimal (i.e., of the order of ~RT). Dotted lines denote interactions with the C-subunit of PKA. cAMP is shown as solid black circles. R_AB_ refers to the PKA-RIα region spanning the two tandem cAMP-binding domains (CBD-A and -B). The cAMP recognition motifs for both CBDs are reported as BBR:cAMP:hinge-lid, where BBR stands for base-binding region. Orange (red) denotes enhanced ms-μs (ps-ns) dynamics. See text in the Discussion for a full explanation. **(B)** Inter-domain contact profile based on the structure of cAMP_2_-bound RIα (91–379) (PDB Code: 1RGS) and SASA changes occurring upon deletion of the 113–232 region (for contacts in the 233–376 segment) or the 233–376 region (for contacts in the 113–232 segment). The boundary between the two deleted regions (i.e., residues 232–233) is based on the observation that the 233–244 region is flexible in RIα (91–244):cAMP (46). **(C)** Profile of RIα (91–379):C contacts as mapped by SASA variations upon deletion of the C-subunit from the structure of the RIα (91–379) R333K:C complex (PDB Code: 2QCS). CBD-A regions experiencing SASA changes in both panels (B) and (C) (i.e., CBD-A residues involved in both CBD-A/B contacts as well as interactions with the C-subunit) are highlighted by dashed boxes.

The comparative NMR analyses of the dynamic conformational equilibria of CBD-A and -B presented here support a “double conformational selection” model for the cAMP-dependent activation of PKA RIα (Fig 6A). In the absence of cAMP the PKA-RIα region spanning the two tandem cAMP-binding domains (CBD-A and -B), denoted as R_AB_, samples four states populating a nearly degenerate free-energy landscape, in which each CBD accesses both C-binding competent (“inactive”) and cAMP-binding incompetent (“active”) conformations with comparable populations, as shown in Fig 2. In the absence of cAMP, such active versus inactive sampling of each CBD occurs independently of the adjacent CBD, due to the absence of significant inter-domain interactions, as shown in Fig 3A–3D. Out of these four nearly degenerate states, the “inactive-inactive” state exhibits the highest affinity for the C-subunit, as both domains are primed for binding C and the inter-domain helical region is also available for interacting with C. The CBD-B affinity for the C-subunit is known to be lower than that of CBD-A, but weak interactions between CBD-B and the C-subunit are still sufficient to drive the conformational equilibria towards the inactive state due to the apo R_AB_ near degeneracy.

The free energy near-degeneracy of apo R_AB_ is eliminated upon binding of two equivalents of cAMP, which select the active state in each CBDs (“active-active” state) and promote CBD-CBD interactions through the lid capping exerted by W260, which is essential to preserve the integrity of the whole CBD-A/B interface, as show in Fig 4A and 4B. The CBD-A/B interface stabilized by cAMP contributes to the dissociation of the R:C complex, because selected CBD-A/B contacts overlap with the R:C interface, as revealed by the SASA difference analysis of Fig 6B and 6C (dashed rectangles). In addition, the inter-domain interactions stabilized by cAMP result in bending of the C-helix, thus further reducing the affinity of R_AB_ for the PKA catalytic subunit (C), which prefers an elongated unbent C-helix [13]. Overall, the emerging evidence suggests that cAMP contributes to the R:C dissociation through three distinct but concurrent mechanisms: *(a)* selection of active conformation of CBD-A, which exhibits low affinity for C; *(b)* selection of active conformation of CBD-B, which further reduces the affinity for C; *(c)* stabilization of inter-domain interactions that are incompatible with the R:C interface.

Mechanism (a), i.e., intra-CBD-A conformational-selection, significantly contributes to the activation of PKA by cAMP, given the high affinity for C of CBD-A in the inactive state [43,44]. However, the contributions of (b) and (c) are essential to explain why CBD-B significantly lowers the *K*_*a*_ for the cAMP-dependent activation of PKA by more than order of magnitude from 1 μM to 80 nM [36]. Although the deletion of CBD-B does not appreciably perturb the affinity of R for C [43], mechanism (b), i.e., intra-CBD-B conformational-selection to stabilize the active state of CBD-B, is still pivotal to promote the inter-CBD interactions, which contribute to the release of C through mechanism (c). Interestingly, even in the presence of cAMP the free energy of inter-domain interaction is marginal compared to the free energy of cAMP binding, as CBD-B deletion does not significantly reduce the free energy of unfolding of CBD-A [36,45–47] or of cAMP-binding to CBD-A [16,18,48,49]. Hence, the “closed” inter-domain topology of R_AB_:cAMP_2_, in which W260 from CBD-B caps cAMP in CBD-A, exists in a dynamic equilibrium with minor populations of an “open” active-active state, in which inter-domain coupling is negligible (Fig 6A). Since intra-domain conformational selection in R_AB_ relies primarily on cAMP binding with only negligible contributions from domain-domain interactions (Fig 4C and 4D), in such an “open” topology of R_AB_:cAMP_2_, which is mimicked by the W260A mutant, the active state selection within each CBD is not compromised, i.e., mechanisms (a) and (b) are still effective, but mechanism (c) is not activated.

A critical feature of the double conformational selection model proposed here to explain the functional role of CBD-B (Fig 6A) is the near free energy degeneracy of the states sampled by apo R_AB_. This degeneracy is quite unique of PKA RIα, as it was not observed for other structurally homologous CBDs, such as those of the hyperpolarization and cyclic nucleotide activated (HCN) ion channels, for which conformational selection relies on apo equilibria that are skewed towards the inactive (auto-inhibited) state with typically low affinity for cAMP [39,50]. This observation provides a possible explanation as to why both CBDs of PKA RIα bind cAMP with higher affinity than HCN, in spite of the structural homology among eukaryotic CBDs. The higher cAMP-affinity exhibited by PKA versus HCN provides a basis to understand the reduced off-rate for cAMP, explaining why cAMP release from PKA RIα would become the rate-determining step of the kinetics of cAMP signal termination by phosphodiesterases (PDEs) in the absence of direct PDE-PKA RIα interactions [51–53].

Furthermore, our investigation revealed marked differences in dynamics also among homologous CBDs in their cAMP-bound forms. For example, in both CBD-A and -B of PKA RIα the adenine base of cAMP is sandwiched by β-strands 4 and 5 (Base Binding Region or BBR) on one side and on the other side by an aromatic side chain lid (i.e., W260 in CBD-A and Y371 in CBD-B), which is part of hinge helices that are C-terminal to the β-subdomains of the respective CBDs. Despite the fact that this structural pattern of cAMP recognition through the BBR and hinge helix-lid motifs is conserved in both CBDs, the BBR:B is significantly more dynamic in the ps-ns timescale than the BBR:A, while the hinge:A is significantly more dynamic in the ms-μs timescale than the hinge:B (Fig 6A). The hinge:A dynamics explain why CBD-A in RIα tolerates, without major affinity losses, cAMP analogues with bulky substituents at the adenine C8 better than anticipated based on the narrow pocket observed in the static structure [54]. In general, these observations corroborate the notion that structurally homologous domains may still exhibit marked differences in dynamic profiles that affect affinities and recognition [55], opening new opportunities for the design of selective ligands [56] that target specific eukaryotic CBDs.

The proposed model (Fig 6) also provides an initial framework to dissect the molecular mechanism underlying disease related PKA RIα mutants. Several mutations reported for PKA RIα CBDs have been linked to the Carney complex and to Acrodysostosis and result in either de-regulation or over-regulation of PKA kinase activity [57–59]. Based on the model of Fig 6, it will be critical to evaluate how such mutations perturb the apo and the cAMP-bound inhibitory equilibria of both CBDs. Perturbations in the apo active versus inactive equilibria lead to changes in cAMP-affinities up to three-orders of magnitude [60], while perturbations in the holo inhibitory equilibria will modulate the inter-CBD interaction which is formed preferentially in the holo/active-holo/active state. Considering the competition between inter-CBD and R:C interactions (i.e., mechanism [c], mentioned above), the modulation of the inter-CBD interaction is expected to in turn alter the *K*_*a*_ for the cAMP-dependent activation of PKA. In general, it is anticipated that the model proposed in Fig 6 will assist in rationalizing how PKA dysfunctional dynamics results in dysregulation of PKA and disease.

## Materials and Methods

### Protein Expression and Purification

All PKA RIα constructs as well as the PKA C-subunit were expressed and purified according to previously published protocols [16,32]. The W260A mutant was prepared by site-directed mutagenesis.

### General NMR Spectroscopy

All NMR spectra were recorded at 306K, unless otherwise specified, using a Bruker AV 700 spectrometer equipped with a TCI cryo-probe and processed with NMRpipe [61] employing linear prediction, unless otherwise specified, and a resolution enhancing 60° shifted sine squared bell window function. All spectra were analyzed with Sparky [62] using Gaussian line-fitting. Assignments were obtained either through triple-resonance 3-D experiments (i.e., HNCO, HNCA, HN(CO)CA, CBCA(CO)NH and HNCACB, in a TROSY-version for high MW constructs) [63,64] and/or through spectral comparisons, if no ambiguities were present. The secondary structure probabilities were determined using the secondary chemical shifts via the PECAN software [65]. Other NMR experiments are discussed below.

### Chemical Shift Analysis

Uniformly ^2^H,^15^N-labeled PKA RIα (91–379) and (91–244) were concentrated to 100 μM in the NMR buffer (50 mM MOPS, pH 7.0, 100 mM NaCl, 10 mM MgCl_2_, 5 mM DTT, with or without 1 mM cAMP, 0.02% sodium azide, and 5% ^2^H_2_O). The C subunit-bound RIα (91–379) and (91–244) complexes were prepared with 1 mM AMP-PNP in the NMR buffer as previously described [16]. Transverse-relaxation optimized spectroscopy (TROSY) 2-D experiments with 80 (t_1_) and 1,024 (t_2_) complex points and spectral widths of 31.82 ppm and 15.94 ppm for the ^15^N and ^1^H dimensions, respectively, were recorded with 12 scans and a recycle delay of 1.70 s. Sensitivity enhanced ^15^N-^1^H hetero-nuclear single quantum coherence (HSQC) spectra with 128 (t_1_) and 1,024 (t_2_) complex points and spectral widths of 31.82 ppm and 15.94 ppm for the ^15^N and ^1^H dimensions, respectively, were recorded with eight scans and a recycle delay of 1.0 s. The ^1^H and ^15^N carrier frequencies were set at the water resonance and at the centre of the amide region, respectively. The C-subunit and cAMP-bound RIα(91–379) forms served as the reference states in the CHEmical Shift Projection Analysis (CHESPA) to evaluate the position of the inhibitory equilibria in the apo RIα(91–379). The combined chemical shifts (CCS), fractional activation, and cos(θ) values were calculated as previously described [37].

### Relaxation Measurements

Uniformly ^15^N-labeled PKA RIα (119–379) wild type and W260A mutant were concentrated to 100 μM in 50 mM MES (pH 6.5), 100 mM NaCl, 5 mM DTT, 2 mM EDTA, 2 mM EGTA, 2 mM cAMP, 0.02% sodium azide and 5% ^2^H_2_O. The R_1_ relaxation rates were measured with water flipback and sensitivity enhanced pulse sequences using relaxation delays of 100 (×2), 200, 300, 400(×2), 500, 600, 800, and 1,000 ms [38]. The R_2_ measurements were acquired using CPMG relaxation delays of 8.48, 16.96, 25.44, 33.92, 42.4, 50.88, 59.36, 76.32, and 93.28 ms with an offset and duty cycle compensated ^15^N R_2_ CPMG pulse sequence with an inter-180° pulse delay of 0.9 ms [38]. Both R_1_ and R_2_ experiments were acquired as pseudo 3-D datasets with recycle delays of 1.5 s. The NOE spectra were collected as a set of ten replicas. The acquired spectra were then co-added in the time domain prior to Fourier transformation. The R_1_ and R_2_ relaxation rates were determined by using cross peak fit heights in Sparky. The errors on the R_1_ and R_2_ rates were estimated from the Gaussian distributed random noise. The errors on the NOE values were gauged based on the standard deviation between fit heights in the replicate spectra. The ^15^N relaxation data were mapped into reduced spectral densities as previously described [66]. The reduced spectral density values in the absence of internal motions were computed through hydrodynamic simulation using the HydroNMR software [67,68] and the cAMP_2_- and C-bound structures (PDB Codes: 1RGS and 2QCS, respectively, but without C-subunit for comparison with the values from 1RGS). An atomic element radius of 2.8 Å was used in all hydrodynamic simulations. The ^15^N relaxation experiments were complemented by H/D exchange data based on HSQC spectra acquired as previously described [32] using the wild type and the W260A mutant RIα (119–379) construct. For this purpose, the protein was concentrated to 100 μM in 50 mM MES (pH 6.5), 100 mM NaCl, 5 mM DTT, 2 mM EDTA, 2 mM EGTA, 0.02% sodium azide, and 5% 2H_2_O. The cAMP bound sample was prepared by adding an excess 100 μM cAMP in the buffer.

## Supporting Information

S1 Data Raw data for Figs 2–6. (XLSX)

## Abbreviations

BBR: base-binding region
C: catalytic subunit
cAMP: cyclic adenosine monophosphate
CBD-A and -B: cAMP-binding domains
CCS: combined chemical shifts
CHESPA: CHEmical Shift Projection Analysis
MD: molecular dynamics
NMR: Nuclear Magnetic Resonance
PBC: phosphate binding cassette
PDEs: phosphodiesterases
PKA: Protein Kinase A
R: regulatory subunit
SASA: solvent accessible surface area
SAXS: small-angle X-ray scattering
WT: wild type
